# Are we adequately preparing the next generation of physicians to prescribe exercise as prevention and treatment? Residents express the desire for more training in exercise prescription

**Published:** 2016-10-18

**Authors:** Kara Solmundson, Michael Koehle, Donald McKenzie

**Affiliations:** 1 Department of Family Practice, University of British Columbia (UBC) School of Medicine; 2 Division of Sports Medicine, Department of Family Practice, University of British Columbia (UBC) School of Medicine

## Abstract

**Background:**

Physical activity (PA) is a key intervention for chronic disease, yet few physicians provide exercise prescription (EP). EP is an important component in larger strategies of reducing non-communicable disease (NCD). Our objective was to assess Family Medicine Residents (FMR) knowledge, competence, and perspectives of EP to help inform future curriculum development.

**Methods:**

A 49-item cross-sectional survey was administered to 396 University of British Columbia FMR. Residents’ EP knowledge, competence, attitudes/beliefs, current practices, personal physical activity levels, and perspectives of training were assessed using, primarily, a 7-point Likert scale.

**Results:**

The response rate was 80.6% (319/396). After eliminating 25 that failed to meet the inclusion criteria, 294 were included in the final analysis. The majority 95.6% of FMR reported EP as important in their future practice, despite having low knowledge of the Canadian PA Guidelines (mean score 1.77/4), low self-reported competence prescribing exercise as prevention (mean score 13.35/21), and rating themselves “somewhat incompetent” prescribing exercise to patients with chronic disease (mean score 11.26/21). FMR believe PA is integral to their patients’ health (98.0%), sedentary behaviour is harmful (97.9%), and feel a responsibility to discuss PA with patients (99.7%). Few FMR (14.9%) perceived their training in EP as adequate and 91.0% desire more.

**Conclusions:**

FMR report EP is important, yet do not perceive they are sufficiently prepared to provide EP. In future curricular development, medical educators should consider residents’ low knowledge, competence, perceived program support, and their expressed desire for more training in exercise prescription.

## Introduction

Physical inactivity is the direct cause of 5 million global deaths annually, and is one of four key modifiable risk factors the World Health Organization (WHO) implore health care practitioners to target in the effort to reduce the pandemic of non-communicable disease (NCD).^1^,^2^

Physical activity (PA) is an effective form of treatment and prevention in over 25 chronic conditions^3^,^4^ and evidence-based Canadian physical activity guidelines (PAG) have been designed to guide PA and optimize the health of all Canadians. It has been reported that if Canadians achieved the PAG level of 150 minutes of moderate to vigorous physical activity (MVPA) per week, the premature death rate of the Canadian population would decrease by 30%, with significant reductions in cardiovascular disease (30%), stroke (25%), osteoporosis (25%), hypertension (20%), diabetes (20%), colon cancer (20%), and breast cancer (14%), in addition to improvements in mental health and quality of life.^3^,^5^–^7^ Despite the irrefutable health benefits of PA, Canadians are insufficiently active. Fifty-one percent of Canadian adults self-report attaining 150 min of MVPA per week,^8^,^9^ yet Canadian Health Measures accelerometer data indicate that only 15% of Canadian adults actually achieve this target.^10^ Similarly, objective accelerometer data indicate that only 7% of Canadian children and youth accumulate their age specific PAG of 60 minutes of MVPA per day, required for their best health and development.^11^

Increasing patient and population levels of physical activity (PA) is essential to address the ballooning health and economic consequences of chronic disease, and physicians have been identified as key catalysts in the solution.^12^–^15^ Physician prescribed exercise has been shown to be cost-effective,^16^–^20^ with a number needed to treat (NNT) of only 12,^17^ in comparison to a NNT of 50 for smoking cessation^21^ performed in conjunction with successful smoking cessation campaigns. However, despite these data, the harms of inactivity, and the benefits of PA, few Canadian physicians prescribe exercise, with a large study reporting only 15.8% of Canadian family physicians (FP) provided written EP.^22^ Barriers to exercise prescription in clinical practice include lack of time, lack of remuneration, and lack of knowledge, training, and skills in exercise prescription. The most frequently reported barrier is the significant lack of education and competence in EP and it is reported across the medical training continuum, from medical students to experienced clinicians.^23^–^26^ Canadian medical students^27^,^28^ and Canadian family physicians^22^,^23^,^29^ have identified training in exercise medicine as deficient and have reported that PA curriculum, included over the course of their medical training, would be both beneficial and desired.

However, few medical schools include exercise medicine in their curriculum.^30^–^36^ American studies indicate that 13% of medical schools in the US offer instruction in PA, 6% have core coursework, and 87% of schools offer no curriculum in exercise medicine whatsoever.^32^ Within medical education there has been an identifiable gap in exercise medicine training, with 64% of medical school deans reporting educating trainees in PA was their responsibility, yet most believed only 10% of their graduates were competent in exercise prescription.^31^ This is starting to change, albeit slowly, in specific institutions, such as the integration of PA into all four years of medical school curriculum at the University of South Carolina^37^, or the establishment of the Institute of Lifestyle Medicine through Harvard Medical School.^38^ However, overall exercise medicine has historically been marginalized in the face of competing interests and topics vying for coverage in medical curriculum.

Interestingly, the importance and need for preparing physicians to discuss and prescribe exercise to patients is widely recognized outside the medical community. The education, training, and clinical practice of EP has been identified and included in global strategies and numerous national policies as a key tactic in the larger strategy of addressing the unprecedented health and economic burdens of chronic NCD.^12^–^14^,^18^,^39^–^42^ In a report on the economic impact of the American obesity crisis, The Bipartisan Policy Centre published a call to action recommending that “nutrition and physical activity training be incorporated into all phases of medical education - medical schools, residency programs, credentialing processes and continuing education requirements.”^43^ Similarly, the Canadian Senate’s recently released report, *Obesity in Canada: A Whole-of-Society Approach for a Healthier Canada*, makes the recommendations: (1) “to encourage improved training for physicians regarding diet and physical activity” and (2) “to promote the use of physician counselling (in Physical Activity), including the use of prescriptions for exercise.”^44^

Family physicians (FP) are well positioned to serve a fundamental role in the promotion of PA to their patients, and play an integral role in improving both patient and population health. FP are identified by patients as a trusted and expected source of health information,^45^,^46^ service a large proportion (80–94%) of the Canadian population,^47^,^48^ care for patients of all ages, provide chronic disease management and continuity of care, and see patients on average 3.1 visits/year.^47^,^48^ FP possess an intimate knowledge of their patients’ health and life circumstances, enabling them to directly discuss the benefits of PA specific to their patients’ health and comorbidities. Likewise, FP can discuss the harms of remaining physically inactive, assist patients in the development of their specific health goals, and assist patients in identifying and overcoming their personal barriers to being active.

This study was designed to address several substantial gaps in the exercise medicine and education literature. Most of the existing studies in EP have been conducted in the medical student or practicing physician populations. There is a notable paucity of EP studies in residency, and limited studies of EP in Canadian medical education, with no prior studies, to our knowledge, of exercise prescription in the Canadian family medicine resident population.

For the following reasons, FMR are an ideal population to educate in exercise medicine and to be future providers and advocates of EP: FMR have (a) a declared area of interest of primary care; (b) a fundamental knowledge base from medical school to build upon; (c) protected academic time to acquire new knowledge and skills; (d) a high volume of patient encounters and therefore opportunity to apply and refine these new skills; and (f) they are malleable as trainees, such that training programs, attending physicians, preceptors, and, potentially, examination scenarios have substantially more influence on their behaviours during residency than following graduation.

To more effectively address the gaps in the literature and to advance the collective knowledge in EP in medical education we designed the present study to assess FMR knowledge, competence, attitudes/beliefs, current PA counselling and EP practices, personal PA levels, perspectives on the importance of EP in their future clinical practice, and perception of their training in EP. We did this to identify key factors to consider in future training interventions.

### Objectives

To assess Family Medicine Residents’:

- Knowledge of the Canadian Physical Activity Guidelines (PAG)
  ○ Awareness of the PAG
  ○ Content of the aerobic PAG for adult and children
  ○ Content of the strength PAG for older adults
  ○ Physical inactivity as a risk factor for mortality (as per WHO data)
- Self-perceived competence in exercise prescription skills:
  ○ Conducting a clinical assessment prior to exercise engagement (as needed)
  ○ Prescribing aerobic exercise
  ○ Prescribing strength exercise
    - in two distinct patient populations:
      ○ Healthy patients
      ○ Patients with pre-existing chronic NCD
- Attitudes and beliefs:
  ○ The importance of PA in their patients’ current health
  ○ Their interest in prevention vs. treatment
  ○ The harms of sedentary behavior on patients’ health
  ○ The effectiveness and credibility of their counselling in relation to their personal exercise and fitness
  ○ Their perception of physician responsibility in PA promotion to patients
  ○ Their perception of program encouragement to practice physically active lifestyles
  ○ Their perception of residency program support/encouragement of residents’ physical activity
- Current PA counselling (PAC) and exercise prescription (EP) practices:
  ○ The frequency in which they provide PAC
  ○ The frequency in which they provide EP
  ○ Their perceived confidence in their skills to prescribe exercise
  ○ Their perceived success at getting patients to start exercising
  ○ Their perceived importance of prescribing physical activity to patients as part of their future medical practice
- Personal PA levels:
  ○ The amount of light, moderate and vigorous PA in which they currently engage
  ○ The amount of strength activity in which they engage
  ○ The amount of time spent sitting on a typical work day and day off
  ○ Their current level of PA compared to before and during medical school
  ○ Their perceived importance of their own personal PA
  ○ Their perceived control over their PA
- Perceptions of training in PA counselling and EP for health, prevention and treatment of disease:
  ○ Training in EP they have received
  ○ Training in EP desired

## Methods

### Setting and participants

Inclusion criteria was all first- (R1) or second-year (R2) FMR actively registered within the department of Family Medicine at the University of British Columbia (UBC) in the two-year family medicine program. Three cohorts of FMR, including the incoming first year (class of 2015), graduating second year (class of 2013), and residents midway through their training (class of 2014), were eligible, a total of 396 possible FMR participants.

### Survey instrument

The survey incorporated established, validated tools such as the International Physical Activity Questionnaire (IPAQ) used to determine residents’ current levels of PA, as well as relevant questions from previous peer-reviewed studies. Factors previously reported as associated with PAC or EP were included in the survey tool, which helped inform the main categories assessed: knowledge of the PAG, self-perceived competence in EP, attitudes and beliefs, current PAC and EP practices, personal PA levels and perspectives of training. To further advance our collective knowledge in exercise medicine and EP in medical education, additional variables identified as important, yet not previously assessed, were developed and incorporated into the survey instrument. These additional questions were designed to address gaps in the literature such as: (1) competence prescribing *exercise as treatment to patients with pre-existing chronic disease*, as well as prevention to healthy patients; (2) competence *prescribing strength or resistance exercise* to patients, as well as aerobic exercise; and (3) assessing personal *sedentary time on both a typical workday and day off*. These additional questions provide more specific data about and greater detail on EP in FM residency training, which then yield a more comprehensive evidence base to inform future curricular development. All questions were assessed independently by three clinicians who are credentialed in family medicine and sports and exercise medicine, two of whom are also exercise physiologists. Questions were tested in an open format on a representative population of recently graduated FMR to facilitate feedback. Comments were carefully reviewed and questions were modified accordingly, following discussion and agreement by the expert panel. Based on the feedback from the open format, the original questions of height and weight, which initially had been included to calculate participants’ body mass index, were modified to the less sensitive wording, “would you describe yourself at a healthy body weight?” The final 49-item research tool was pilot-tested by 10 family medicine physicians and recent FMR to ensure face validity, clarity, and timing, prior to administration to the target audience of UBC FMR (see Supplemental content with questionnaire).

### Study design and protocol

The research tool was administered via the Canadian web-based platform, Fluid Surveys, electronically to residents, through the UBC FM list-serve sent by the FM program administrator. A cover letter described the purpose and nature of the study, and included details of consent, the potential risks and benefits of participation, confidentiality, privacy, and contact information for both the lead researcher and the UBC office for the rights of research participants. The voluntary nature of participation and details of consent were clearly outlined within the cover letter, as was the implied consent to participate, if they chose to click on the hyperlink to the survey. Additional on-site opportunities were available for residents to complete the survey on e-tablets during three program-wide events: research day, new resident orientation, and a program wide academic day. The survey design and study were approved by the UBC Behavioral Research Ethics Board.

### Data analysis

Data were analyzed using IBM SPSS statistical version 21 software using descriptive statistics. Incomplete questionnaires were eliminated by applying the objective criterion that the importance of exercise prescription in future practice (question #18), must have been answered to be included in the analysis.

Questions were designed to facilitate responses on seven-point (7-pt) Likert scales. The 7-pt scale was chosen specifically, as it allows analysis as either a categorical measure, or as a continuous measure as Dr. Geoff Norman, one of the world’s leaders in medical education research methodology, has comprehensively reviewed.^49^ The main 7-pt Likert scale used was: 1-pt strongly disagree, 2-pt disagree, 3-pt somewhat disagree, 4-pt neutral, 5-pt somewhat agree, 6-pt agree, 7-pt strongly agree. Any variations of the specific responses used on the 7-pt scale (such as 1-much less to 7-much more, 1-highly incompetent to 7-highly competent, and 1-most important risk factor to 7-least important risk factor) are detailed below. Data were analyzed individually for all factors assessed and categorically by collapsing the data categorically into negative, neutral, and positive responses, such that the main 7-pt Likert scale would become disagree (1–3), neutral (4) and agree (5–7). This level of collapse was consistent for all categorical data analysis.

Questions 1 and 2 (Q1 and Q2) inquired about PA levels prior to and during medical school and used the 7-pt scale: 1-much less, 2-less, 3-somewhat less, 4-the same, 5-somewhat more, 6-more, 7-much more. Data were analyzed individually and categorically - less (1–3), the same (4) or more (5–7). Questions 3–11 assessed the level of PA engagement of FMR. Q3–Q10 were comprised of the International Physical Activity Questionnaire (IPAQ) and Q11 assessed the level of strength PA of FMR. Overall, metabolic levels of PA were calculated for FMR in accordance to the updated International Physical Activity Questionnaire (IPAQ) scoring protocol.^50^ Metabolic equivalent (MET) minutes per week were calculated based on the intensity, duration, and frequency of the activity. Using the IPAQ standardized METs for walking (3.3 METs), moderate intensity (4.0 METs), and vigorous intensity (8.0 METs), moderate PA performed for 30 minutes, 5 days a week would be calculated as 4.0*30*5 = 600 MET-min/week. A total MET-min/week was calculated for each FMR as follows: (walk MET*min*days) + (moderate METs*min*days) + vigorous METs*min*days) = Total MET-min/week.^50^

Questions 12 and 13 assessed the importance and control over FMR personal exercise using the main 7-point Likert scale.

Current PA counselling (Q14) and EP practices (Q15) were assessed on an ordinal scale that was not an equal interval scale, chosen specifically to better detail residents’ current practices. Data are reported as a frequency table.

Attitudes and beliefs (Q19–23) were assessed by the main 7-pt Likert scale and thus, analyzed individually and categorically as described. Additionally, an overall attitude and belief score was calculated as the sum of five questions (Q19–Q23), for a possible mean score range of 5–35, using inverse scoring for question Q20 “prevention is NOT as interesting to me as treatment.”

Questions 24 and 25 pertain to the role of program support in PA using the main 7-point scale.

Awareness of the PAG (Q26) elicited a “yes, no, or unsure” response, and knowledge of the recommended levels of PA specific to the adult (Q 27), pediatric (Q28), and older adult (Q29) populations were determined by correctly identifying the guideline. Physical inactivity as a risk factor in chronic disease deaths (Q30) was assessed by ranking physical inactivity among other established risk factors on the 7-pt Likert scale - 1-most important/greatest contribution to 7- least important/least contribution to chronic disease deaths. Responses were assessed individually and a total knowledge score was calculated by the sum of correct responses (excluding awareness); this was done by allocating one point per correct response for each of the three PAG questions and for physical inactivity being rated among the top 4 risk factors according to WHO data^51^–^53^ for a maximal knowledge score of 4 (range 0–4). Individual scores were assessed and used to calculate a mean knowledge score of FMR.

Questions 31–36 assessed self-perceived exercise prescription competence on the 7-point Likert scale: 1-highly incompetent, 2-incompetent, 3-somewhat incompetent, 4-neutral, 5-somewhat competent, 6-competent, 7-highly competent. The 6 questions were designed to assess three skills of exercise prescription: conducting a clinical assessment prior to exercise engagement; prescribing aerobic exercise and prescribing strength exercise, as prevention (to healthy patients) and treatment (to patients with pre-existing chronic disease). These data were analyzed in three ways: (i) each skill individually (range 1–7); (ii) as a composite competence score calculated as the sum of the three exercise prescription skills in (a) healthy patients (range 3–21) compared to the same three skills to (b) patients with NCD (range 3–21); and (iii) as an overall competence score, by the sum of all six exercise prescription skills (range 6–42).

Residents’ perspectives of the education and training in EP received (Q37) and desired (Q38) were assessed on the main 7-pt Likert scale (1-strongly disagree to 7-strongly agree). Data were analyzed individually and categorically in the same manner used throughout the analysis - disagree (1–3), neutral (4) and agree (5–7).

## Results

There was an 80.6% response rate - 319 of 396 eligible FMR participated in the study. Twenty-five questionnaires were eliminated, due to failure to satisfy inclusion criteria. Specifically, 24 were incomplete and one was not an R1 or R2 in the two-year residency program at the time of the study. After eliminating 25 of the 319 responses, 294 (74%) surveys were included in the final analysis.

The mean age of respondents was 30 years (range = 25–54, SD = 5.1) with 64.9% female and 35.1% male. All 14 training sites across British Columbia (BC) participated, providing representation from distinct geographical areas of BC, which included costal, northern, and interior communities. The large cohort of FMR participants included rural, urban and aboriginal FM training programs, and involved residents providing care for diverse populations and communities throughout BC. There was an even distribution by stage of training: 37.5% beginning FM residency (graduating class of 2015), 33.8% midway (class of 2014), and 28.7% completing (class of 2013) Family Medicine residency. Respondents reported previous exposure or training in exercise medicine prior to residency as follows: sports medicine course (24.8%), preventative medicine course (12%), undergraduate course in human kinesiology (15.3%), coaching certification (12.6%), exposure to extensive exercise medicine curriculum during medical school (1.4%), or other forms of training related to exercise medicine (6.8%). The majority of respondents (78.3%) reported they were a “healthy body weight”, but less than half (45.7%) reported they felt physically fit.

### Residents’ perception of the importance of exercise prescription in their future practice

The majority of respondents (95.6% (n=281)) indicated exercise prescription will be important in their future practice (5–7 on 7-pt Likert scale) with individual responses detailed in Table 1.

### Personal physical activity levels

Residents report being less physically active during residency than during medical school and prior to medical training (Table 2). Only 51.9% of FMR meet Canadian PAG level of 150 min of moderate to vigorous PA (MVPA) per week, according to the validated International Physical Activity Questionnaire (IPAQ). When total metabolic equivalent minutes per week (MET-min/week) were calculated (see methods for IPAQ scoring protocol and MET-min/week calculations) 18.1% of residents were highly active (>1500 MET-min/week), 33.8% were moderately active (>600 MET-min/week), and 48.1% were insufficiently active (<600 MET-min/week), to attain the guideline level of PA. Only 24.5% of FMR satisfy the Canadian strength PAG of regularly performing resistance exercise twice per week.^54^

Family Medicine Residents spent more time sitting on a typical workday (6.59 hours +/− 3.35 hours) than on a day off (4.93 hours +/− 2.65 hours).

Physical activity was reported to be personally important (Likert 5–7) to 96.3% (n=283) of FMR, however, they perceived lower levels of control (Likert 5–7) over their personal physical activity (74.9%, n= 220). Interestingly, 58.5% (n=172) of FMR indicated high importance (7/7-pt Likert) of their personal exercise, yet only 17.0% (n=50) of FMR reported high control over it.

### Current physical activity counselling and prescription practices

During a typical primary care office encounter, FMR reported counselling patients in physical activity more frequently than prescribing exercise (Table 3). FMR reported higher confidence (Likert 5–7) (62.2%, n=183) in their EP skills than perceived success in getting their patients active (Likert 5–7) (28.3%, n=83), although few residents indicated they felt either highly (7/7-pt Likert) confident (11.6%, n=34), or successful (3.8%, n=11) in exercise prescription.

### Attitudes and beliefs

FMR have highly positive attitudes and beliefs regarding PA. Residents report (Likert 5–7) they believe PA is integral to their patients’ health (96.6%, n=284), that sedentary behaviour is harmful (96.3%, n=283), and 86.5% (n=250) disagree with the statement “prevention is not as interesting to me as treatment.” FMR believe they will be able to provide more credible and effective counselling if they personally exercise and stay fit (94.9%, n=279), and 98.3% (n=289) of FMR believe physicians have a responsibility to promote PA to their patients. Nearly all FMR, (95.9%, n=282) believe their academic programs should encourage them to lead physically activity lifestyles, yet this belief contrasts with the lack of exercise-related support FMR report receiving from their programs (50.0%, n=147) (Table 4).

### Knowledge of the physical activity guidelines

The proportion of respondents who reported being familiar with the Canadian PAG (33.7%, n=94) was greater than the proportion who could correctly identify the recommended level of 60 minutes of moderate to vigorous PA (MVPA) per day for children (23.4%, n=64) and the recommendation for older adults (>65 years) to engage in strength exercise twice per week (31.0%, n=85). However, roughly half of the residents (52.2%, n=144) were able to correctly identify the more widely promoted PAG of 150 minute of MVPA for Canadian adults (Appendix 1).^3^,^55^,^56^ Most residents (70% n=205) correctly identified physical inactivity as one of the top four causes of mortality in accordance with WHO data.^51^ A total knowledge score was calculated for each resident, allotting 1-point for each correct response, out of a maximal score of four. FMR demonstrated low overall knowledge with a total mean score of 1.70 (+/−1.16).

### Self-reported competence in exercise prescription

Residents reported greater competence in EP as primary prevention to healthy patients (HP), compared to prescribing exercise to patients with pre-existing non-communicable diseases (NCD). FMR indicated they felt most competent prescribing aerobic exercise and least competent prescribing strength, which was the same for both patient populations. Mean scores for all three exercise prescription skills assess: clinical assessment, aerobic EP and strength EP, in both patient populations are detailed in Table 5. Residents overall exercise prescription score for all six skills was (24.58 (+/− 0.83) out of 42).

### Family Medicine Residents’ perception of their training in exercise prescription

Only 14.9% (n=42) of FMR perceive (Likert 5–7) that they have received adequate training in exercises prescription. This low number starkly contrasts with the 91% (n=252) of residents who report a desire (Likert 5–7) for additional training in exercise prescription (Table 6).

## Discussion

This study highlights that 95.6% (n=281) of UBC Family Medicine residents believe that exercise as a medical intervention will be important in their future practice, which is greater than the 53–79% of Canadian and American medical students who reported relevance of PA prescription to their practice.^24^,^27^,^28^,^57^–^59^ The higher importance of EP in the present study may be attributable to several factors, including that the majority of studies reported to date have been conducted in the medical student population, which by comparison are a relatively undifferentiated cohort. In contrast, FMR have chosen primary care and are generally more interested in prevention than their medical colleagues who have chosen an acute, tertiary care based medical discipline. Both interest in prevention and primary care have been reported in the medical student population to be positively associated with a higher perceived relevance of physical activity counselling.^57^–^59^

### FMR physical activity levels

UBC FMR are more active than American residents,^60^,^61^ slightly more active than the Canadian population,^62^ but are less active than Canadian and American medical students, which is consistent with the reports in the literature that medical students are more physically active than residents.^27^,^28^,^58^ There is increasing awareness of the harms attributable to physical inactivity and, more recently, the harms of sedentary behaviour. Public health campaigns heralding “sitting is the new smoking” is increasing awareness. However, this is the first study to assess the differences in sedentary time of residents, both while on and off duty. It is worth noting that FMR engage in over 90 minutes less sedentary time on a day off than a typical workday, which suggests when FMR have more control over their schedule, they are more active.

### Active doctor = active patient?

Physically active physicians and medical students have been reported to provide more PA counselling to patients^28^,^29^,^57^,^63^ yet the limited studies of residents in the literature has not upheld this relationship.^60^ Our data similarly did not follow the pattern of Active Doctor = Active Patient, which likely reflects the diminished PA levels of residents, rather than the relationship between physicians’ health behaviours and counselling practices. UBC FMR engage in less PA than they did during and prior to medical school, and report high importance, yet low control over their personal exercise. Interestingly, this contrast of importance vs. control FMR perceive over their personal PA, parallels the high importance FMR attribute to program support of residents being physically active, compared to the low support they perceive receiving. Few U.S. internal medicine residents report high self-efficacy to engage in sufficient PA, and with their low levels of PA, their suitability to be role models in PA for patients has been challenged.^60^,^64^,^65^ It’s been reported that “enjoyment and self-efficacy of exercise,” of internal medicine residents were predictive factors of residents’ success with exercise counselling.^60^ Our data highlight the discordance between FMR low current levels of PA and the high importance, yet low control they perceive over their personal exercise. This raises the concern that if UBC FMR have low self-efficacy in their own ability to engage in exercise, how effective will they be in successfully engaging patients in exercise?

There is a marked discrepancy between UBC residents’ current EP practices and their stated importance of EP in future practice, which may in part be due to the deficiencies in knowledge, competence, and training, which our data indicate are low. Therefore, it is possible that UBC FMR are not currently providing exercise prescription because they simply do not yet feel competent doing so. There are additional factors specific to the resident population that can have a profound impact on current EP behaviour, including preceptor leadership and program support, or lack there-of. Tsui indicated that both American internal medicine residents and attending physicians had low competence prescribing PA, and suggested future training interventions should target not only residents, but include parallel education of staff physicians in exercise prescription.^66^ With large studies of practicing Canadian family physicians reporting low competence^23^ and low frequency in providing detailed exercise prescription^22^ to patients, it is certainly plausible that residents may lack essential leadership in preceptors, attending physicians, and programs in exercise prescription.

### Current physical activity counselling and exercise prescription practices

The frequency of PA discussion and physician delivered PA advice to patients decreases as the level of detail and requisite knowledge required increases. Generalized statements (“physical activity is good for you”) are most common, specific PA counselling is less common, and detailed exercise prescription, the least.^22^,^23^,^67^,^68^ The frequency of providing patients with these levels of intervention directly parallels the confidence of the physicians in PA counselling and exercise prescription, and UBC residents’ PA counselling and EP practices follow this pattern.

Substantial efforts are being made to increase the frequency of discussion of PA in patient encounters. Exercise is Medicine Canada (EIMC) is part of the EIM global health initiative, established in over 43 countries, designed to increase exercise counselling and prescription in the healthcare setting.^69^ EIMC’s mission is “to provide national leadership in promoting physical activity as a chronic disease prevention and management strategy to improve the health of Canadians” and strongly advocates using “Exercise as a Vital Sign.” While UBC family medicine residents’ current exercise counselling and prescription behaviours are within the ranges reported in the literature,^70^–^72^ they fall well short of the EIMC goal of asking “every patient in every encounter,” and present an opportunity to increase the frequency of PA dialogue in patient encounters.

### Awareness of the physical activity guidelines

Few studies have assessed awareness of PAG, and our findings show that only one-third of residents have knowledge of the Canadian PAG. This is consistent with results in other settings: only 40% of UK medical students and 12% of American internal medicine residents were aware of their respective guidelines.^36^,^60^ An earlier study of Canadian family physicians identified the lack of clear PAG as an important barrier to EP and concluded specific guidelines would assist physicians in providing exercise counselling to patients.^23^ Subsequently, accumulating scientific data have informed the evidence derived PAG’s, which are consistent with the PAG of the WHO and are acknowledged globally as important health targets. Our findings of lack of awareness and knowledge give cause for concern particularly given the rapidly increasing prevalence of NCD in Canada. Physician knowledge of the principles of exercise medicine and familiarity with the recommended levels of PA for individuals is an essential component of any strategy that seeks to improve population health.^35^,^36^,^73^,^74^ Others have identified this as an important area of training deficiency within current educational programs.^35^,^36^,^66^,^74^

### Overall knowledge of residents

Both the individual and overall low knowledge scores highlight the inadequacy of the education currently provided to trainees. These data suggest we are not providing FMR with sufficient education in exercise medicine, nor providing them with the basic knowledge they require to effectively advise patients with respect to PA. This may reflect the lack of formal instruction in exercise medicine, which is not unique to Canada and is evident in medical training at all stages. Researchers from the United Kingdom reported that medical trainees are exposed to a mean of 109 hours (range 18–336) of curriculum time allocated to pharmaceuticals in contrast to an average of 4.2 hours devoted to physical activity issues,^75^ which is likely not dissimilar to Canadian Medical education.

Of particular concern from the present study is the finding that over three-quarters of FMR underestimated the daily hour of physical activity recommended for children’s health and development. Given the increasing alarm which is accompanying the rising rates of childhood obesity and sedentary behaviour in Canada, it is surprising and disconcerting that FMR are unaware of the level of PA required for children to meet current guidelines.

Interestingly, the majority of residents overestimated the PAG recommendation of strength exercise in the older adult population of at least two times per week. This may reflect the instruction residents receive in the importance and role of strength and balance exercises, currently embedded in the fall prevention and osteoporosis units of the curriculum. This finding might also indicate that when specific PA or exercise recommendations are included in the curriculum, there is uptake.

### Competence in exercise prescription

Our findings among FMR of low levels of perceived competence regarding exercise prescription are similar to those identified in studies of graduating medical students,^24^ residents^65^,^66^ and practicing family physicians.^22^,^23^ It is not surprising that medical personnel consistently report low levels of knowledge and competence in exercise prescription given the minimal exposure to these concepts at all stages of their professional training.

UBC FMR report they feel most competent prescribing aerobic exercise and least competent prescribing strength exercise, in both healthy patients and NCD patient populations. With FMR most frequently engaging in aerobic PA (51.9%), and few personally engaging in strength exercise (24.5%), our data follow Abramson’s findings that clinicians are more likely, and feel confident to, prescribe exercise(s) of a similar type in which they personally engage.^76^ These data suggest an experiential component to EP curriculum may warrant consideration for inclusion in future program development.

Not surprisingly, FMR report greater competence advising healthy patients in physical activity as primary prevention compared to providing EP to those with chronic disease. To our knowledge this is the first study to examine perceived competence prescribing exercise to patients’ with pre-existing chronic NCD, in addition to healthy patients. This distinction is important as the incidence and prevalence of chronic disease continues to grow. According to a recent report by the Public Health Agency of Canada, 3 of out 5 Canadians over age 20 already have a chronic disease, with 4 out of 5 at risk for developing a chronic condition.^77^ FMR rated themselves as “somewhat incompetent” on the 7-pt Likert scale in each of the three skills of EP for patients with pre-existing NCD. It is essential that physicians receive fundamental training in exercise medicine to ensure they have the knowledge base to safely and effectively prescribe exercise to *all* their patients, regardless of their patients’ age or comorbidities. Physician EP knowledge, skills and competence will become increasingly critical in the context of Canada’s growing epidemic of chronic disease.

### Limitations and strengths

Limitations of this study include risks of social acceptability, response and non-response biases that may be partially mitigated by the large (80.6%) response rate, which would include residents inherently less interested in the topic and ensure that the data are representative of all UBC residents. Although the cross-sectional design does not allow comparisons over time, the study was not evaluating an intervention (it was proximal to that stage) and the design was appropriate for the objectives of the present study. Participants were exclusively UBC FMR, which may limit the generalizability of the results beyond this population. However, UBC family medicine is a diverse program with coastal and interior sites, spread across Northern, Central, and Southern BC and includes urban, rural and aboriginal programs. Therefore, despite being under the common UBC FM umbrella, all 14 sites are distinct and participation of residents from each site helps ensure a rich diversity of program design and representation, which may further mitigate the degree of specificity. With the paucity of research in EP in residency, and our awareness of no prior study in EP in a Canadian Family Medicine residency population, the findings of this study can contribute important data to the evolving body of literature of exercise medicine in medical education. Therefore, it may be valuable to other family medicine residency programs, primary care residency programs (pediatrics, internal medicine), or relevant for other stages of medical training, as comparative data or a framework to assist in program evaluation and development.

### Conclusions

Our findings underscore the need for enhanced education and training in exercise prescription during family medicine training at UBC and perhaps other Canadian post-graduate training programs. FMR have low knowledge, skills, and self-reported competence in EP, despite having strong beliefs in the benefits of PA and reporting EP will be important in their future practice. FMR perceive their medical training in EP as inadequate and indicate their expressed desire for more. Our data suggest that tomorrow’s family physicians are entering practice with insufficient preparation in EP – a deficiency that is all the more urgent, given the rising prevalence of NCD and the unprecedented health and economic implications of chronic disease on Canadian society.

In the context of the growing pandemic of physical inactivity and chronic disease, the need to educate, train and empower physicians in exercise prescription is critical. We need to provide FMR, the next generation of FM physicians, with the skills and knowledge to feel confident discussing PA and competent providing individualized EP to all of their patients. Our study underscores the current deficiencies of exercise prescription in family medicine residency training. Our findings contribute important data to an evolving evidence base of curriculum development,^73^,^78^ highlight specific factors that merit consideration in the development of future EP interventions in FM residency training, and warrant the attention of medical educators.

## Figures and Tables

**Table 1:** Family medicine residents’ perceived importance of exercise prescription in their future practice

	Strongly Disagree % (n)	Disagree % (n)	Somewhat Disagree % (n)	Neutral % (n)	Somewhat Agree % (n)	Agree % (n)	Strongly Agree % (n)
“Prescribing physical activity to my patients will be an important part of my FUTURE medical practice”^1^	-	-	0.3 (1)	4.1 (12)	15.6 (46)	42.2 (124)	37.8 (111)

1 Responses scored on the 7-point Likert scale: 1- Strongly Disagree, 2-Disagree, 3-Somewhat Disagree, 4-Neutral, 5-Somewhat Agree, 6-Agree, 7-Strongly Agree

**Table 2:** FMR current PA levels, %(*n*)

Compared to:	Less Active (1–3)	The Same (4)	More Active (5–7)
During Medical School^1^	49.1 (144)	22.2 (65)	28.7 (84)
Prior to Medical Training^2^	64.8 (190)	16.7 (49)	18.4 (54)

1 Defined as first two years – pre-clerkship/pre-ward duties

2 Defined as two years prior to medical school

3 Responses scored on the 7-point Likert Scale of the amount of exercise residents currently engage in: 1-Much Less, 2-Less, 3-Somewhat Less, 4-the Same, 5-Somewhat More, 6-More, 7-Much More

**Table 3:** Family medicine residents’ physical activity counselling and prescription practices (n=293)

During a typical office encounter:	I counsel^1^ patients on physical activity ___ of the time % of residents (n)	I prescribe^1^ physical activity to patients ___ of the time % of residents (n)
Never <5%	1.4 (4)	16.7 (49)
Rarely 5–20%	10.6 (31)	22.5 (66)
Occasionally 21–40%	20.8 (61)	18.4 (54)
Sometimes 41–60%	21.4 (63)	17.1 (50)
Frequently 61–80%	25.9 (76)	17.4 (51)
Nearly Always 80–95%	13.7 (40)	4.4 (13)
Always >95%	6.1 (18)	3.4 (10)

1 Specify physical activity “dose” = frequency, intensity, time + type

**Table 4:** Family medicine residents’ attitudes, beliefs and perceptions of physical activity

Attitude/Belief	Strongly Disagree (SD) % (n)	Disagree (D) % (n)	Somewhat Disagree (sD) % (n)	Neutral (N) % (n)	Somewhat Agree (sA) % (n)	Agree (A) % (n)	Strongly Agree (SA) % (n)	Likert 5–7 sA+A+SA
“I believe that regular PA is integral to my patients’ current health”	-	0.3 (1)	0.3 (1)	1.4 (4)	5.1 (15)	25.9 (76)	65.6 (193)	96.6 (284)
“Prevention is NOT as interesting to me as treatment”^1^	40.8 (120)	34.7 (102)	9.5 (28)	4.8 (14)	4.1 (12)	1.7 (5)	2.7 (8)	85.0 (250)
“I believe sedentary behaviour is harmful to my patients’ health”	1.0 (3)	0.7 (2)	-	0.3 (1)	3.7 (11)	27.2 (80)	65.3 (192)	96.3 (283)
“I will be able to provide more credible and effective counselling if I exercise and stay fit”	0.3 (1)	0.7 (2)	0.7 (2)	1.7 (5)	10.2 (30)	40.1 (118)	44.6 (131)	94.9 (279)
“I believe physicians have a responsibility to promote physical activity to their patients”	-	-	-	0.3 (1)	3.7 (11)	34.0 (100)	60.5 (178)	98.3 (289)
“Residency programs should encourage their residents to practice physically active lifestyles”	-	-	0.3 (1)	1.0 (3)	5.4 (16)	26.9 (79)	63.6 (187)	95.9 (282)
“My residency program encourages residents to exercise and be physically active”	4.4 (13)	9.2 (27)	11.6 (34)	22.8 (67)	23.8 (70)	17.7 (52)	8.5 (25)	50.0 (147)

1 Inverse scoring when calculating categorical score: SD+D+sD

**Table 5:** Family medicine residents’ self-reported competence prescribing exercise to healthy patients and patients with chronic non-communicable disease

Exercise Rx Skill	Competence^1^,^2^	Healthy Patients		Patients with chronic noncommunicable disease (NCD)	

		%	n	%	n
Conducting a clinical assessment (clear for exercise)	Incompetent	29.3	82	50	140
	Neutral	12.1	34	15	43
	Competent	58.6	164	34.6	97
	Total mean (max score 7) (95% CI)	4.46 (4.29–4.63)		3.69 (3.52–3.86)	

Prescribing aerobic exercise (type, frequency, intensity, duration)	Incompetent	19.4	54	43.6	122
	Neutral	12.9	36	13.9	39
	Competent	67.6	188	42.5	119
	Total mean (max score 7) (95% CI)	4.75 (4.59–4.91)		3.95 (3.79–4.11)	

Prescribing strength or resistance exercise (type, frequency, repetitions, sets)	Incompetent	37.8	105	53	148
	Neutral	14.0	39	15.1	42
	Competent	48.2	134	31.9	89
	Total mean (max score 7) (95% CI)	4.15 (3.96–4.34)		3.61 (3.45–3.77)	

EP Competence (sum of 3 skills^3^) to Healthy Patients and NCD Total mean (max score 21) (95% CI)		13.35 (12.90–13.80)		11.26 (10.81–11.71)	

1 Responses scored on the 7-point Likert scale: 1-highly incompetent, 2-incompetent, 3-somewhat incompetent, 4-neutral, 5-somewhat competent, 6-competent, 7-highly competent;

2 Categories from the 7-point Likert scale: 1–3=Incompetent, 4= Neutral, 5–7=Competent;

3 Sum of competence scores across three skills of exercise prescription: clinical assessment, prescribing aerobic and prescribing strength exercise

**Table 6:** Family medicine residents’ perceptions of exercise prescription (EP): the importance of EP in their future practice, EP training received and EP training desired

Question	Response^1^	% (n)
“Prescribing physical activity to my patients will be an important part of my FUTURE medical practice”	Disagree	0.3 (1)
	Neutral	4.1 (12)
	Agree	95.6 (281)

“I have received an adequate amount of education/training on physical activity counselling and exercise prescription for health, prevention and treatment of disease during my family medicine residency training^2^”	Disagree	61.8 (173)
	Neutral	23.2 (65)
	Agree	14.9 (42)

“I would like to receive more education/training on physical activity counselling and exercise prescription for health, prevention and treatment of disease”	Disagree	1.4 (4)
	Neutral	7.6 (21)
	Agree	91 (252)

1 Responses from the 7-Point Likert scale were categorized as disagree, neutral and agree as follows: Disagree=1–3 (Strongly Disagree, Disagree, Somewhat Disagree); Neutral=4; Agree=5–7 (Somewhat Agree, Agree, Strongly Agree)

## Appendix 1 Family Medicine Residents’ knowledge of the Canadian Physical Activity Guidelines and physical inactivity as a risk factor for mortality

**Table:**

Physical Activity Guideline Question	Percent (%)		Frequency (n)	

Are you aware of the Canadian Physical Activity Guidelines?				
Yes	33.7		94	
No	44.1		123	
Unsure	21.2		62	

**Adults (18–64 yo) should accumulate at least ___ minutes of moderate intensity physical activity each week:**				

60	3.3		9	
90	11.2		31	
120	16.7		46	
150	52.2		144	
180	8.3		23	
210	8.3		23	

**Children (5–17 yo) should accumulate at least ___ minutes of moderate intensity physical activity each week:**				

30 min × 5 days = 150	18.2		50	
30 min × 7 days = 210	20.1		55	
45 min × 5 days = 225	8.0		22	
45 min × 7 days = 315	8.8		24	
60 min × 5 days = 300	21.5		59	
60 min × 7 days = 420	23.4		64	

**Older adults (>64 yo) should perform strength training:**				

0 days/week – it’s contraindicated in this population	0		0	
At least 1 day/week	2.2		6	
At least 2 days/week	31.0		85	
At least 3 days/week	60.2		155	
There is no evidence specific to strength training in this population	3.3		9	
There are no guidelines around strength training in this population	3.3		9	

**Rank the following risk factors in Descending order of importance to chronic disease deaths according to World Health Organization data: From 1 (Most important/Greatest contribution to 7 (Least important/Least contribution)**				

**Risk Factor**	**Mode**	**Mean**	**(95% CI)**	**Rank**
HTN	4	4.11	(3.89–4.33)	4
Smoking	1	2.41	(2.19–2.63)	1
Impaired glucose	5	4.36	(4.16–4.56)	5
Physical activity	2	3.42	(3.21–3.63)	2
Overweight/Obesity	3	3.46	(3.25–3.67)	3
Hyperlipidemia/High Cholesterol	6	5.35	(5.15–5.55)	7
Excessive alcohol use	7	4.96	(4.74–5.18)	6

**Total Knowledge Score = Sum of 4 Knowledge questions (not awareness)**				

	**Percent (%)**		**Frequency (n)**	
0/4	12.6		37	
1/4	32.1		94	
2/4	32.4		95	
3/4	18.4		54	
4/4	4.4		13	
Mean knowledge score (95% CI)	1.77 (1.65–1.89)			
